# An important Norwegian contribution to the study of the bursae of the upper and lower extremities

**DOI:** 10.3109/17453674.2010.506633

**Published:** 2010-10-08

**Authors:** Vladimir Musil, Christoffer V Selnes, Aleksander T Falck, Lars Sandve, Siamek Shekarchi, Bruce O'Donnell, David Kachlik

**Affiliations:** ^1^Centre of Scientific Information, Third Faculty of Medicine, Prague, Czech Republic; ^2^Institute of Information Studies and Librarianship, Faculty of Philosophy and Arts, Prague, Czech Republic; ^3^Department of Anatomy, Third Faculty of Medicine, Charles University in Prague, Prague, Czech Republic

## Abstract

We present a critical analysis of the monograph of A.S.D. Synnestvedt (1869) “En anatomisk beskrivelse af de paa over- og underestremiteterne forekommende Bursae mucosae”. The analysis was completed using anatomical information from the historically oldest publications dealing with the bursae of the extremities: Albinus (1734), Monro (1788), Rosenmüller (1799). We are of the opinion that Synnestvedt's publication is important, not only historically but also as a source of information for recent medical practitioners. Synnestvedt's monograph has a wealth of literary citations, unambiguous opinions of seasoned anatomists regarding the structure and function of the synovial membrane, and detailed descriptions of dissections he performed on fetal and adult cadavers. The information in this publication may enhance the diagnosis of bursopathies and enthesopathies of the extremities.

The modern systematic diagnostics and therapy of some bursopathies and enthesopathies began before the end of the nineteenth century, particularly with the synovial vaginae of the foot (Hartmann 1896), Baker's cyst (Rauschning 1979), achillodynia (Albert 1893, Rössler 1895), and others. Even today, the investigation of ethiopathogenesis and diagnosis and therapy of disorders of tendinous insertions, tendon synovial sheats, and the bursae still go on, particularly in sports medicine. However, it is not generally known that the exact anatomical description of the bursae and tendinous sheaths was established in the latter half of the nineteenth century in Norway.

The purpose of this article is to provide detailed information regarding an important and almost forgotten Norwegian monograph published 140 years ago. This monograph remained almost unknown amongst anatomists and clinicians until 2006. Two of the authors (VM and DK) discovered, in the literary vault of the First Faculty of Medicine at Charles University in Prague, the work of Andreas Svane Dick Synnestvedt during their search relating to the clinical anatomy of the retrocalcaneal bursa (Kachlik et al. 2008a,b,c, Kachlik et al. 2010). The monograph had been published in 1869 and was entitled “En anatomisk Beskrivelse af de paa Over- og Underextremiteterne forekommende Bursae mucosae, stottet paa egne lagttagelser og ledsaget af Tegninger efter udforte Praeparater”. In order to understand the contents, we enlisted the aid of 4 Norwegian students and their Irish colleague from the Third Faculty of Medicine in Prague (CS, AF, LS, SS, and BO'D). The 4 students were asked to translate the contents of the monograph from Norwegian to English.

When the translation was complete, it became apparent to us how important the contents of this publication were, not only in terms of its historical origin but also for the valuable scientific information it contained. We decided to compare this work with both the oldest and the most recent data regarding the morphology and terminology of the bursae of the upper and lower extremities. Furthermore, we believed that through this comparison we could estimate its importance to the morphological disciplines and its possible value in clinical practice.

## Methods

We used the Norwegian original along with the English counterpart of Synnestvedt's monograph (Synnestvedt 1869), two of the most important monographic works of the eighteenth century pertaining to the anatomical description of the bursae (Monro 1788, Rosenmüller 1799), and all versions of the official anatomical terminology (Final Report 1933, Donáth 1969, FCAT 1998). In all of the aforementioned sources, we compared the quantity of bursae of the upper and lower extremities that were mentioned, along with their description and classification. The data obtained were also evaluated in terms of their possible use in modern clinical practice.

## Results

### Anatomical descriptions of bursae during the 18^th^ century

According to Monro (1788), refers to the first name, not the surname. It has no place here] from a historical point of view the first detailed and systematical comment regarding the bursae of the extremities had been made by Bernard Albinus (1697–1770) in Leyden, 1743. In his work “Historia musculorum hominis” (Albinus 1734), he described 16 pairs of bursae in both extremities (Table) without any detailed description.

**Table:** Summary of the numbers of bursae mentioned and described in all monographs and official anatomical terminologies discussed

	Albinus	Monro	Rosenmüller	Synnestvedt	B.N.A.	B.R	I.N.A.	P.N.A.	T.A.
	1734	1788	1799	1869	1895	1933	1935	1955	1998
Σ UE	4	33	35	48	21	14	20	15	14
Shoulder	3	9	11	17	8	6	9	9	9
Elbow	1	3	6	16	9	6	8	6	5
Hand	–	– (+21 Vag)	– (+18 Vag)	15	4	2	3	–	–
Σ LE	12	37	39	73	39	30	41	32	30
Hip	5	13	14	21	14	12	14	13	12
Knee	7	9	8	29	15	12	17	14	13
Foot	–	2 (+13 Vag)	1 (+16 Vag)	23	10	6	10	5	5
Total	16	70	74	121	60	46	61	47	44

More than 50 years later, Alexander Monro Jr. (1733–1817), an anatomist from Edinburgh, published a 60-page monograph on the systematic anatomy of the bursae entitled “A description of all the bursae mucosae of the human body” (Monro 1788). Monro described 70 pairs of bursae on the extremities, with 34 of them being identified as tendinous synovial vaginae (Table). This monograph was also important in that it contained over 40 citations of works from previous distinguished authors who had been researching bursae before (e.g. Albinus, Havers, Heister, Hunter, Malpighi, and Weitbrecht).

Johann Christian Rosenmüller (1771–1820) published his large monograph “Alexandri Monroi icones et descriptiones bursarum mucosarum corporis humani” (Rosenmüller 1799). The book consisted of 108 pages and 15 plates of illustrations. This work was not just a mere translation of Monro's book, but rather an unbiased and innovative publication that incorporated Latin and German text in parallel. Furthermore, Rosenmüller described 71 pairs of bursae on the upper and lower extremities, including 34 tendinous synovial vaginae (Table). Although he did not expand on the amount of previously identified bursae, he was able to make the distinction between the proper bursae (bursae vesiculares) and the tendinous synovial sheaths (bursae vaginales).

**Figure 1.**
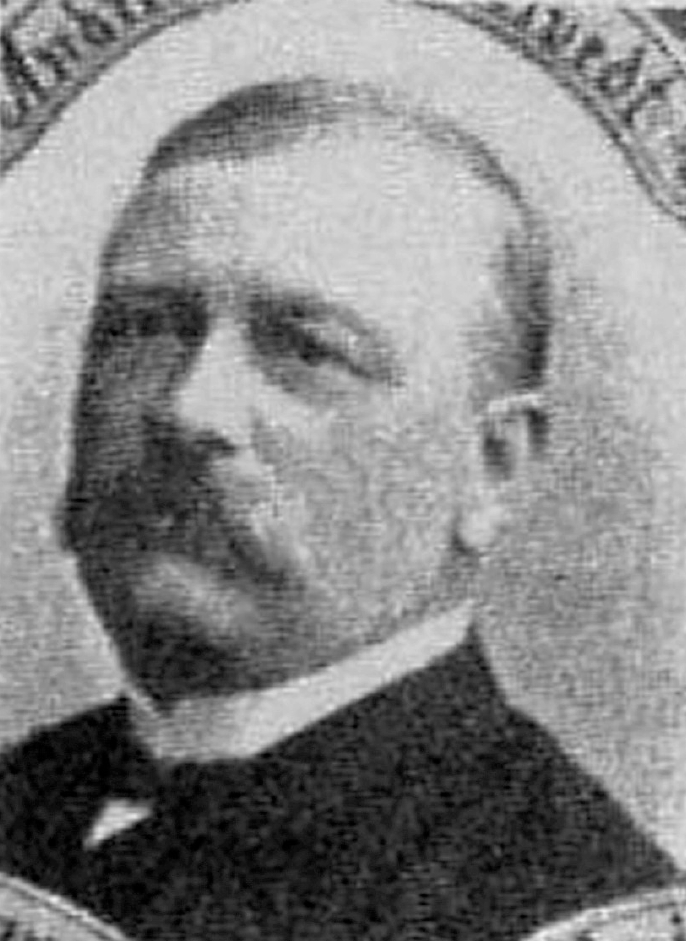
Andreas Svane Dick Synnestvedt.

Andreas Svane Dick Synnestvedt (Figure 1) was born on August 5, 1844 in Bolsø, Norway. He graduated from the Trondhjem Kathedralskole in 1861. He then studied medicine at the University of Christiania, where he graduated on May 30, 1870. His mentor, Professor Voss, presented him with the topic of his thesis “the anatomical description of the bursae of the upper and lower extremities”. Synnestvedt finished his thesis and successfully defended it against the anatomical council in 1868. It was the opinion of the council that his work was of such a high standard that he should be awarded the Skeldrup's Gold Medal and that his work would be published in the form of a monograph, the costs of which were to be covered by the University. The monograph (Synnestvedt 1869) became an obligatory part of the curriculum of the Faculty of Medicine in Christiania (Storm-Mathisen 2008). After graduation, Synnestvedt spent the rest of his life practicing medicine. During 1870, he held several posts in Trøndheim (obstetrics, military medicine), Levanger (district medical officer), and Skogn (chief surgeon). From 1870–1883 he was the chief municipal officer in Vaerdelen; he was a district medical officer in Rollag (1883–1890) and held the same position in Inderøen (1890–1909). His contribution to Norwegian medicine as well as his social standing among his colleagues was illustrated in his obituary in “Morgenbladet”: “S. was a man whose warmth and personal kindness made him a lot of friends; he was highly thought of everywhere he practiced as a doctor. At social gatherings he was an extremely popular man, and he had a particularly important role in the student society”.

### A brief description of Synnestvedt's monograph

**Figure 2.**
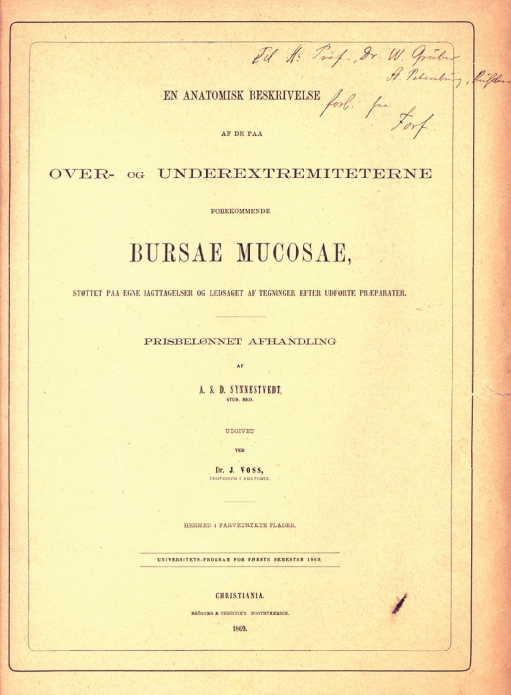
Title page of Synnestvedt's monograph.

**Figure 3.**
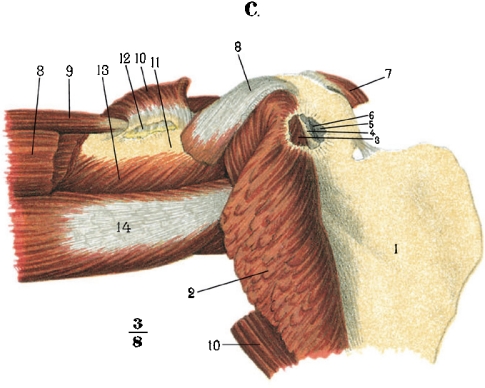
An example of the bursae in the shoulder region. 3: subscapular bursa; 12: subtendinous bursa of teres major.

**Figure 4.**
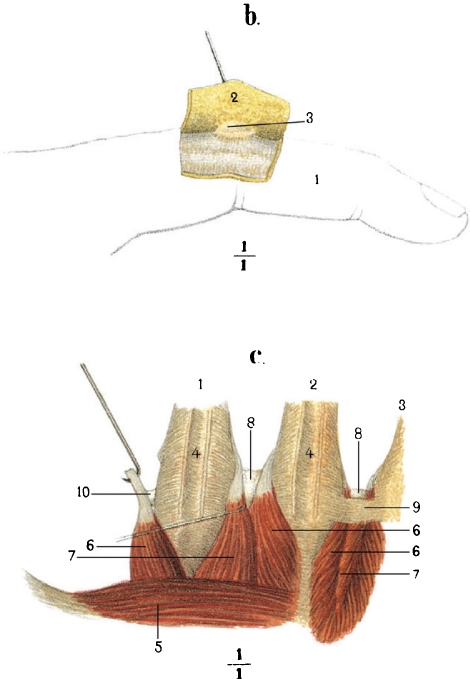
b. Index finger: 3: subcutaneous mucous bursa. c. 1–3: metacarpals II–IV; 8: intermetacarpophalangeal bursae.

**Figure 5.**
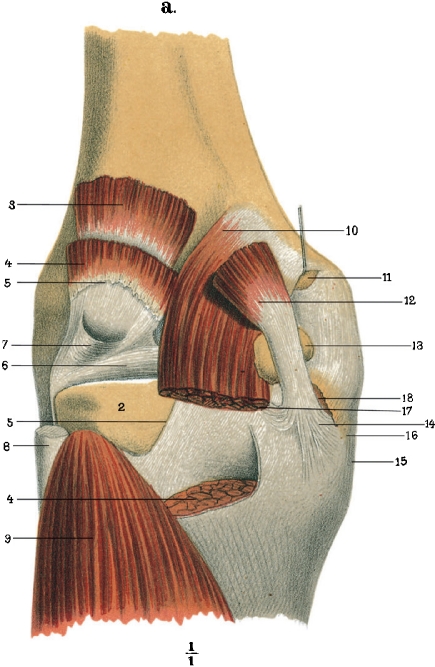
Depiction of same postgenual bursae. 11: medial subtendinous bursa of gastrocnemius; 13: semimembranoso-gastrocnemial bursa; 18: semimembranous bursa, prolongating in front of and behind the anterior part of semimembranosus.

**Figure 6.**
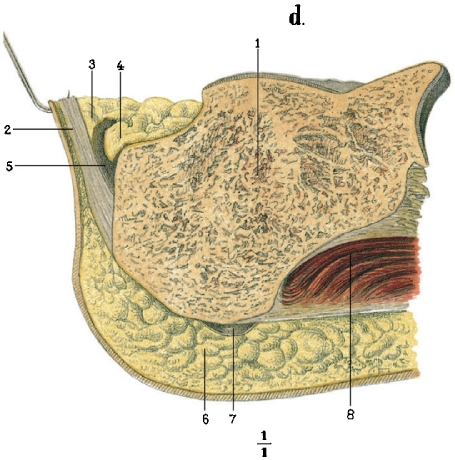
Sagittal section through the heel region. 2: calcaneal tendon; 3: adipose tissue, covering the superior wall of the bursa; 4: adipose fold, roaming freely in the bursa; 5: retrocalcaneal bursa; 7: subcalcaneal bursa.

This publication (Figure 2) includes 88 pages of text and 4 pages of color illustrations (Figures 3–6). The introduction includes the summary of the Evaluation Committee (Academic Collegium) of the Medical Faculty in Christiania dated September 16, 1868: “…It has the character of a planned and independent work and is distinguished in its order, preciseness, and completeness. The accompanying drawings are realistic. We find this thesis to be not only worthy of Skjeldrup's Gold Medal, but we have also taken the liberty of recommending that the university should finance the publication of the material as it is missing from the anatomical literature”. On the next page the author described how his study was based on the dissection of 106 partially intact extremities obtained from the Department of Anatomy. He mentioned the general lack of appropriate human dissection material, which prevented him performing a larger study, and also mentioned the lack of access to older literature dealing with the anatomy of the bursae.

In the Prologue (13 pages) that followed, the author established the distinction between mucous and serosal membranes, based on their different histological structures. To the category of serosal membranes he placed peritoneum, pleura, pericardium, synovial membranes of joint capsules, and those of the bursae and the tendinous synovial vaginae. From this large and complex group of structures, he categorized a further 2 groups of morphologically distinct structures, i.e. the tendinous “synovial vaginae” and the bursae proper. In the case of the bursae proper, Synnestvedt decided to designate it using old and incorrect—yet still accepted—terminology: “bursae mucosae”. During his continued description of bursal anatomy, Synnestvedt mentioned different types of synovial folds and villi; furthermore, he contradicted the belief of previous authors that these structures work as glands (“glandulae mucilaginosae Haversianae”) and produce the synovia, which enters the cavities of the bursae via small ducts inside the synovial folds. His belief was that the contents of these folds produce the adipose tissue and blood vessels responsible for the production of the synovia and are structurally, chemically, and functionally different from the mucous ones. Synnestvedt proceeded to provide an insight into the chemical analysis of the synovial fluid, which is interesting from a historical analytical chemistry point of view but unimportant in clinical anatomy nowadays. Another interesting observation made by Synnestvedt revolved around his use of fetal material and the prenatal presence or absence of some bursae. The case was a 7-month-old fetus and through examination, Synnestvedt agreed with the opinions of previous authors that while the deep bursae develop during the prenatal period, the subcutaneous bursae appear postnatally and are dependent on the physical activity of the individual.

The individual anatomical description of the bursae was presented in the 64 subsequent pages of text. Synnestvedt identified and described 48 pairs of bursae on the upper extremity and 73 other ones on the lower extremity (Table). The tendinous synovial vaginae were excluded from the observation. Synnestvedt paid a substantial degree of attention to the localization, size, and topography of the bursae, with practically no clinical comments. He also focused on the variability of the bursae and the presence in some cases of secondarily acquired ones. In these cases, Synnestvedt made reference to recent literature, particularly that of the latter half of the nineteenth century, and compared his own findings with them. From the authors cited, it is important to mention the founder of modern histology Albert von Kölliker (1817–1905), Joseph Hyrtl (1810–1894) and Wenzel Gruber (1814–1890). Both Hyrtl and Gruber began their professional carriers at the Medical Faculty of Charles University in Prague, but their most successful years were spent in Vienna and St. Petersburg, respectively. These anatomists had dedicated a large proportion of their careers to the study of the bursae, and thus it was evident why Synnestvedt cited them many times. Gruber (1857) had at this point published 15 papers regarding the anatomy of the bursae, particularly that of the knee region. In his 97 references to historical and recent anatomical texts, Synnestvedt did not mention the work of Walter Heineke. This indulgence can be forgiven, considering that Heineke's work was only published shortly before Synnestvedt's (Heineke 1868).

### Bursae in the official anatomical terminologies

During the latter quarter of the nineteenth century, great efforts were made in the unification of the Latin form of anatomical terminology with more modern ones. The first official version, the B.N.A (Basiliensia Nomina Anatomica), was presented and approved by the Congress of the Society of German-Speaking Anatomists—Anatomische Gesellschaft—in 1895, in Basel, Switzerland (Kachlik et al. 2008d). It was the decision of the Congress to reduce the number of listed bursae to approximately half of those cited, in comparison with Synnestvedt's monograph. The same principle was applied in the “Birmingham Revision” (Final Report 1933) and again in the current version of anatomical terminology (I.N.A.—Ienaensia Nomina Anatomica) published in Jena (Germany) in 1935 (Table). The number of bursae was further reduced by one quarter with the next official publication of international terminology (P.N.A.—Parisiensia Nomina Anatomica, Paris, 1955) (Donáth 1969). This reduction was echoed with the publication of the last variant of the terminology named T.A. (Terminologia Anatomica) in Stuttgart and New York in 1998 (FCAT 1998). The reasoning behind these reductions (Table) was never explained.

Interestingly, in the B.N.A., the official terms “vagina mucosa” and “bursa mucosa” were used. The I.N.A. and T.A. use the acknowledged expressions “bursa synovialis = synovial bursa” and “vagina synovialis = synovial sheath”.

## Discussion

It is evident that this young medical student, through careful research on both adult and fetal objects and extensive literary review, created a truly magnificent anatomical publication. In comparison to previous authors, Synnestvedt's written account was the most extensive, because Monro (1788) dedicated only one quarter of his book to the anatomical description of the bursae, and Rosenmüller (1799) the same, whereas Synnestvedt (1869) dedicated more then three quarters.

In general, the importance of this monograph can be seen in the light of the fact that it correctly describes the common structural features of the synovial membranes of joint capsules, bursae, and tendinous sheaths. Furthermore, it rejects the glandular theory of synovial folds, presents an exact classification and systematic anatomical description of the bursae of the extremities, and does so by using many (then) recent and historical anatomical references.

This publication represents the culmination of classical descriptive anatomy regarding the bursae in the eighteenth and nineteenth centuries. As this book was originally written in Norwegian, it was only cited sporadically (Hartmann 1896, Rauschning 1979, Kachlik et al. 2008a-c). It is also possible that the knowledge of the detailed morphology of the bursae of the extremities, in clinical terms, was viewed as unimportant. Regardless of the reasons why this occurred, there exist a number of reasons in modern society why it is important to re-examine this information:

the symptomatology of bursitis and enthesopathy and their common localizations (shoulder, elbow, hip, knee, and heel) are frequent complaints encountered by modern medical clinicians in the fields of general and orthopedic surgery, rheumatology, sports medicine, and rehabilitation

as we have demonstrated in our example of the heel region (Kachlik et al. 2008c), an enhanced knowledge of the normal anatomy of the bursae can substantially help to improve the differential diagnosis of bursitis and tendovaginitis in many regions.

Thus, we believe that our intentions are just, when through close collaboration, the Departments of Anatomy in Prague and Oslo plan to finish the terminological analysis and re-publication of Synnestvedt's text. We hope that this revised edition will be completed before the end of 2010 and will contain the original Norwegian text along with its English facsimile, and it will be available from the Publishing House of Charles University, Vydavatelstvi Karolinum (www.cupress.cuni.cz). We also believe that the English version of this text will be of value to the anatomical and clinical communities.

We hope that through our efforts, we will to some extent be able to enact the word set forth in Hyrtl's motto (translated from German original): “The opinions of old authors are not valid any more and their historical research is held to be superfluous. Thus, there is no doubt that this kind of research will be considered worthless by many anatomists. But it was not so for me, as I believed that it is more correct to follow—retrospectively and back to its original sources—everything that has any relationship with the historical development of the artistic face of anatomy, to follow the directions that I believed were promising during my life, and this appeared to me to be the only competent way” (Hyrtl 1873).
